# Malnutrition in Hospitalised Children—An Evaluation of the Efficacy of Two Nutritional Screening Tools

**DOI:** 10.3390/nu13041279

**Published:** 2021-04-13

**Authors:** Christina N. Katsagoni, Olga Cheirakaki, Anastasia Hatzoglou, Ourania Zerva, Alexandra Koulieri, Konstantina Loizou, Emmanouela Vasileiadi, Maria Toilou, Kalliopi-Anna Poulia, Meropi D. Kontogianni

**Affiliations:** 1 Department of Nutrition & Dietetics, School of Health Sciences and Education, Harokopio University, 70 El. Venizelou str., 176 71 Athens, Greece; christina.katsagoni@gmail.com (C.N.K.); oxeirakaki@yahoo.gr (O.C.); dp4421226@hua.gr (A.H.); ds21105@hua.gr (E.V.); mtoilou93@gmail.com (M.T.); 2 Department of Nutrition and Dietetics, “Agia Sofia” Children’s Hospital, 4 Thivon and Papadiamatopoulou str., 115 27 Athens, Greece; 3 Department of Nutrition and Dietetics, “P. and A. Kyriakou” Children’s Hospital, Thivon and 13 Livadias str., 115 27 Athens, Greece; rzerva@gmail.com (O.Z.); al_koul92@yahoo.gr (A.K.); knloizou@gmail.com (K.L.); 4 Department of Nutrition and Dietetics, “Laiko” General Hospital of Athens, Agiou Thoma 17, 115 27 Athens, Greece; lpoulia@gmail.com

**Keywords:** malnutrition, nutritional risk, screening tool, paediatrics, PYMS, STAMP, WHO

## Abstract

Nutritional risk screening (NRS) is not yet established in many clinical settings. This study aimed to evaluate the efficacy of two NRS tools; the Paediatric Yorkhill Malnutrition Score (PYMS) and the Screening Tool for the Assessment of Malnutrition in Paediatrics (STAMP), compared to the global dietitians’ clinical judgment. The goal of this study was also to estimate the prevalence of nutritional risk in Greek paediatric patients. Overall, 1506 children, 1–16 years, from paediatric and surgical wards of two Greek hospitals were included. NRS was performed using PYMS and STAMP based either on World Health Organization (WHOGC) or Hellenic growth charts (HGC). The first 907 children were also referred to dietitians who categorized children in low, medium and high nutritional risk according to their global clinical judgment. PYMS, either based on WHOGC or HGC, showed better agreement with dietitians’ feedback (k_PYMS_WHO_ = 0.47; 95%CI: 0.41–0.52, k_PYMS_HGC_ = 0.48; 95%CI: 0.43–0.53) compared to STAMP (k_STAMP_WHO_ = 0.28; 95%CI: 0.23–0.33, k_STAMP_HGC_ = 0.26; 95%CI: 0.21–0.32). PYMS also showed the best diagnostic accuracy compared to STAMP in paediatrics and surgical wards separately. Moreover, the PYMS showed similar sensitivity to the STAMP (WHOGC: 82% vs. 84.4%), but a higher positive predictive value (WHOGC: 58.2 vs. 38.7). Using PYMS, high and medium malnutrition risk was observed at 14.9%, and 13.1% of children, respectively. Almost 28% of hospitalised children were at nutritional risk. Children in hospitals should be screened with effective and feasible NRS tools such as PYMS.

## 1. Introduction

Hospitalised children are at higher risk of developing malnutrition, especially disease associated malnutrition [1,2]. According to ESPEN (European Society of Parenteral and Enteral Nutrition) terminology, malnutrition is synonym to undernutrition and several aetiology-based types of malnutrition exist, such as disease-related malnutrition, which is a specific type of malnutrition, caused by a concomitant disease with or without inflammation [3]. Moreover, according to ESPGHAN (European Society for Paediatric Gastroenterology Hepatology and Nutrition), appropriate nutritional status and malnutrition are difficult to define and assess, due to challenges in differentiating the impact of malnutrition and the disease state on markers of malnutrition and on outcome [4]. Indeed, disease-related malnutrition in children is the consequence of a complex interplay of various etiological factors, including reduced food intake due to anorexia, feeding difficulties or the effects of medications or due to the hyper metabolic state caused by the underlying disease per se [5,6,7]. Based on the opinions of health care professionals who routinely assess and treat children with disease-related malnutrition. ongoing weight loss, increased losses, increased requirements, low intake and high nutritional risk conditions seem to be the most important clinical indicators [8].

Disease-related malnutrition in hospitalised children in Europe has been associated with a significantly prolonged length of hospitalisation and increased complications, such as diarrhea and vomiting, with possible considerable cost implications, and reduced quality of life [5,9]. According to a recent study the subsequent increased healthcare costs due to malnutrition can reach the 80 million euros per year [10]. Unfortunately, disease-related malnutrition of hospitalised children remains a serious problem that is often unrecognized or underestimated by paediatricians and other allied health care professionals [6,11,12]. 

The importance of early identification of disease-related malnutrition risk has been highlighted for many years [4]. However, a thorough clinical and nutritional assessment of malnutrition is time-consuming, often requiring special equipment and trained personnel [13]. Consequently, the nutrition risk screening tools have been proposed to easily and quickly identify patients at risk of malnutrition [14], so they could be thereafter referred to clinical dietitians for a more thorough clinical and nutritional assessment. Although nutritional risk screening has been for many years proposed as a necessary routine practice by many scientific associations [4,15,16], its implementation is not yet established in many clinical settings. The main barriers seem to be lack of time, lack of financial and staff resources, low awareness regarding nutrition and/or because the efficacy of the available tools have not been widely tested [8].

To accurately evaluate nutritional risk, it is important to choose an efficient nutrition risk screening tool [14]. Several such tools are available in the paediatric population [14], while most of them have been designed according to main principles of ESPEN for Malnutrition Screening Tools [14,17]. They consist of questions regarding children’s medical history, dietary intake, nutritional status, as well as alterations in the disease-related nutritional status. Nevertheless, a paucity of research exists on what is considered to be the most appropriate nutrition screening tool to be applied in hospitalised children [14] and validated in different populations, as well as which tool is more efficient in relation to specific disorders or clinical environments [13]. Moreover, high heterogeneity exists over the definition of the gold standard for the validation of screening tools [18].

To the best of our knowledge, such data are sparse in Greece. Therefore, the present study aimed to evaluate the efficacy of two well-known nutrition risk screening tools, namely the Screening Tool for the Assessment of Malnutrition in Paediatrics (STAMP) [19] and Paediatric Yorkhill Malnutrition Score (PYMS) [20] in identifying paediatric patients at nutrition risk in a sample of Greek hospitalised children upon admission, against dietitians’ global clinical judgment. The secondary aim was to estimate the prevalence of malnutrition risk in a random sample of hospitalised children, and hence, to raise awareness for paediatric malnutrition in hospitals.

## 2. Materials and Methods

### 2.1. Subjects

In this cross-sectional evaluation with prospective follow-up study 1506 children from the two biggest paediatric hospitals of Greece in the ATTICA province, i.e., “P. & A. Kyriakou” Children’s Hospital and “Agia Sofia” Children’s Hospital were included. Eligible for enrolment were considered all children that were admitted to paediatric and surgical wards and were expected to be hospitalised for at least one day. 

The children’s parents or their caregivers were fully informed about the study goals and provided written consent before their child’s enrolment in the study. The study protocol was approved by the Scientific Boards of the two Paediatrics hospitals and it was carried out in accordance with the Declaration of Helsinki [21].

### 2.2. Anthropometric Measurements

The children’s age and sex, as well as their underlying disease upon admission and length of hospitalisation were recorded. Anthropometric measurements were performed at the time of admission, with children barefoot and in light clothing, using a standard scale and a portable stadiometer to the nearest 0.1 kg, and 0.1 cm, respectively. For children under 2 years of age, length was measured in a lying down position on a firm surface with their head against a hard board. Body mass index (BMI) was calculated as weight (in kilograms) divided by height (in meters squared) and z-scores were derived of all measurement. To determine underweight both the World Health Organization Growth Charts (WHOGC) [22] and the Hellenic Growth Charts (HGC) [23] that are still partially implemented in the clinical practice, were used. 

Nutritional screening was performed using two already published tools: the STAMP [19] and the PYMS [20]. In specific, STAMP was developed in UK and validated for hospitalised children aged 2–17 years [19] assessing 3 parameters, i.e., the disease effect on nutritional status, the dietary intake and the child’s growth status, namely height and weight. Accordingly, PYMS was developed also in UK and validated for hospitalised children aged 1–16 years [20] and evaluates four components: Current nutritional state (by measuring patient’s BMI), recent weight loss and recent decrease in dietary intake, as well as the possibility of deterioration of patient’s status as a result of current disease. In both tools, a final score is calculated according to which children are classified in three categories: High, medium and low risk of disease-related malnutrition.

The aforementioned tools were completed by the same operator in two ways: (a) Based on the WHOGC; and (b) based on the HGC.

### 2.3. Dietetic Referrals

Apart from the screening questionnaires’ completion, the first 907 recruited children were also referred to clinical paediatric dietitians of the corresponding hospitals to provide their global clinical judgment. The dietetic feedback was based on the principle that the tool could identify the majority of the patients who needed dietetic referral for malnutrition issues in a clinical setting, as also previously implemented in other studies [20,24]. Specifically, dietitians retrieved information from the medical records and combined them with information on recent dietary intake, malnutrition focused physical examination (e.g., oedema, wasting) and basic anthropometric measurements. Based on these data, they categorized children in three categories of nutritional risk (i.e., low, medium, high).

### 2.4. Statistical Analysis

As the high nutritional risk prevalence of paediatric disease related malnutrition in Europe at the time of admission is ranging from 6.1 to 25% [25,26,27] we assumed that the prevalence of high nutritional risk in paediatric populations in our country is around 12%. Using a confidence interval of 98% and a margin of error of 2%, the required sample size of the present study was found to be 1434. 

The normality of variables was checked through Shapiro-Wilk test and graphically through histograms. Continuous variables are presented as median (interquartile range, IQR) as all variables were skewed. Qualitative-categorical variables are presented as absolute (n) and relative frequencies (%). Kappa Value (k) was used to determine the agreement between the assessed tools. In case of complete agreement between tools then k = 1. In order to check the validity of the corresponding tools (i.e., PYMS and STAMP), the nutrition outcome of each tool was initially cross-tabulated with that of dietetic global judgment. Low and medium risk categories of patients were combined to determine the diagnostic values [i.e., sensitivity, specificity, positive (PPV) and negative (NPV) predictive values] of the screening tools compared to the dietetic feedbacks. Then, the aforementioned analyses were also applied to paediatric and surgical wards separately, in order to check tools’ validity based on wards’ type. Once verifying the tool with the best diagnostic accuracy, logistic regression analysis was used to explore whether the corresponding tool’s steps could also predict the malnutrition outcome derived from the dietetic judgments. Kruskal-wallis H test was used to compare the differences between the risk categories of the chosen tool and several characteristics (i.e., anthropometric characteristics of children, length of hospitalisation etc.) across the whole study sample. The tool with the best agreement with the dietetic review was also used to estimate the overall prevalence of the disease related malnutrition risk. A two-sided alpha level of 5% was used to indicate statistical significance. Analyses were performed using the statistical package SPSS (version 21.0 for Windows, SPSS modified April 2020, Chicago, USA).

## 3. Results

Participants’ characteristics are shown in Table 1. Children’s median (IQR) age was 5.7 (3.0–10.5) years. Their median (IQR) body mass index of 16.6 (15.2–19.2) kg/m^2^, whereas about one out of five children reported a recent unintentional weight loss. From the total of 1506 inpatients participated in the present study, 868 (58%) were hospitalised in the paediatric wards and 638 (42%) were hospitalised in the surgical wards. Overall, the main causes of admission were gastrointestinal (GI) track disease and minor surgeries. The first 907 enrolled in the study were also sent for nutritional risk assessment by a hospital dietitian. This subsample did not differ from the whole in terms of age, sex and BMI (Table 1). 

Nutritional screening tools diagnostic accuracy compared to dietitians’ clinical judgment is presented in Table 2. PYMS completed based on WHOGC showed better albeit moderate agreement with the dietitians’ judgment (k_PYMS_WHO_ = 0.47; 95%CI 0.41–0.52) compared to STAMP (k_STAMP_WHO_ = 0.28; 95%CI 0.23–0.33). When the diagnostic ability of the corresponding tools was checked to paediatric and surgical wards separately, PYMS showed again the best diagnostic accuracy compared to STAMP based on WHOGC (paediatric wards: k_PYMS_WHO paediat_ = 0.42; 95%CI 0.36–0.49 vs. k_STAMP_WHO paediat_ = 0.32; 95%CI 0.25–0.38; surgical wards: k_PYMS_WHO surg_ = 0.58; 95 %CI 0.49–0.68 vs. k_STAMP_WHO paediat_ = 0.21; 95% CI 0.13–0.29 (Supplementary Table S1). When HGC were used to determine growth status in children, the diagnostic accuracy of PYMS was again better than that of STAMP’s either in the whole sample (k_PYMS_HGC_ = 0.48; 95%CI 0.43–0.53 vs. k_STAMP_HGC_ = 0.26; 95%CI 0.21–0.32) (Table 2) or in the paediatric or surgical wards, separately (paediatric wards: k_PYMS_HGC paediat_ = 0.42; 95%CI 0.36–0.48 vs. k_STAMP_HGC paediat_ = 0.29; 95%CI 0.22–0.35; surgical wards: k_PYMS_HGC surg_ = 0.61; 95%CI 0.52–0.70 vs. k_STAMP_HGC paediat_ = 0.22; 95%CI 0.14–0.29) (Supplementary Table S1). 

**Figure 1:**
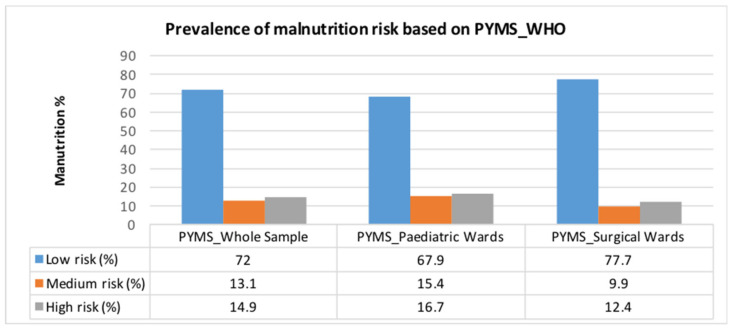
Prevalence of disease related malnutrition risk based on PYMS_WHO in the whole sample (n = 1506) and in Paediatric (n = 868) and Surgical (n = 638) wards separately (PYMS: Paediatric Yorkhill Malnutrition Score; WHO: World Health Organization).

As PYMS was found superior to STAMP, either based on WHOGC or HGC, further analyses were performed in the whole sample of 1506 children based on this screening tool. The overall prevalence of the disease related malnutrition risk in the study’s sample is shown in Figure 1. Medium and high risk was observed in 13.1% and 14.9% of children, respectively. The corresponding percentages in paediatric and surgical wards were 16.7% and 12.4%, accordingly (*p* < 0.001). The results were similar when the tool was implemented based on the HGC *(*Supplementary Figure S1).

Children’s characteristics in relation to the risk categories based on PYMS_WHO are shown in Table 3. Children at high risk of disease related malnutrition compared to those at low or medium risk were younger, with reduced growth, while most of them reported recent weight loss and low food intake. Moreover, length of hospitalisation was greater for those at high risk compared with low risk patients (Table 3). As HGC are still in use in Greek clinical practice, results were also explored with PYMS_HGC and remained unchanged (data not shown).

Nine out of 65 children (13.8%) who were categorized as at high risk from dietitians, were missed from PYMS (i.e., categorized as low or medium risk) (Table 4). However, among missed cases, none of the children was undernourished, based on the BMI of −2 SDSs, while only 1/9 children showed a recent weight loss and 3/9 presented with a low food intake (all *p* < 0.05). In addition, hospitalisation length was lower among missed cases compared to non-missed cases (*p* = 0.002) (Table 4). 

However, each separate step of PYMS_WHO tool significantly and independently predicted the likelihood of nutritional risk, based on dietitians’ clinical judgments, after controlling for age and sex (all *p* < 0.05) (Supplementary Table S2) and this was also true for PYMS_HGC (data not shown). 

## 4. Discussion

The present study aimed to compare two previously published nutrition risk screening tools, namely the PYMS [20] and the STAMP [19], in a Greek paediatric population. Based on the current results, PYMS compared to STAMP presented with the most acceptable levels of sensitivity and specificity against dietitians’ global assessment. PYMS superiority was also independent of the growth charts used in the present study (i.e., WHOGC or HGC) as well as the wards implemented (i.e., paediatric vs. surgical). Therefore, its use in clinical practice is recommended. Using PYMS to estimate the overall prevalence of disease related malnutrition, it was found that 13.1% and 14.9% of children were at medium, and high risk of malnutrition, respectively, and these data should be disseminated in Greek paediatric clinical settings to raise awareness for detecting disease-related malnutrition in a routine way.

Disease-related malnutrition in hospitalised children has several consequences which nowadays are increasingly recognized. However, no agreement has been reached upon the “gold standard” for the assessment of malnutrition risk [28]. Indeed, a recent systematic review [13] reported that there is insufficient evidence to choose one nutritional screening tool over another–comparing Paediatric Nutritional Risk Score (PNST), STAMP, PYMS and STRONG-kids-based on their predictive accuracy. Similar results were shown when comparing STRONG-kids with PNST in another study [29]. Moreover, given that there is a high heterogeneity in the literature regarding the gold standard for validating screening tools, WHO anthropometry measurements have been considered as possible reference combined with nutritional assessment [18]. In the present study, PYMS based either on WHOGC or HGC was compared to dietitians’ global assessment and it was proved to be superior to STAMP. This is in line with the review of Klanjsek et al. [18], in which PYMS was recommended as the most appropriate tool for hospitalised paediatric patients. 

A tool that could stratify with the maximum sensitivity and specificity all children according to their nutritional risk would be ideal [20]. In the present study, the positive predictive value of PYMS was 58% based on WHOGC; much higher than that of STAMP (i.e., 39%). Similar results have been shown in the study of Gerasimidis et al. [20], in which the PPV of PYMS was 44% versus 31% of that of STAMP, as well as in the study of Pars et al. [30], in which PYMS was compared with STRONG-kids (32.6 versus 27%, accordingly). However, 13.8% who were categorized as at high risk from dietitians, were missed from PYMS. Indeed, dietitians’ global assessment might have triggered from other valid reasons (e.g., increased energy/nutrient requirements or increased nutrient losses) [8] that cannot be assessed by a tool. Nevertheless, children’s hospitalisation length was lower among missed cases compared to non-missed cases, suggesting that children may suffer from conditions that could affect children’s future nutritional status.

Furthermore, PYMS was found superior to STAMP when the analysis was performed, either in the whole sample or separately in paediatric and surgical wards. This is of great importance, as the same tool seems appropriate for use in a wide range of patients in clinical practice, making the nutrition screening process less complicated. Furthermore, some of the reported barriers to nutrition screening, such as lack of time [8,13,31] could be overcome if a quick-and-easy screening tool, such as PYMS was adopted universally in both paediatric and surgical wards.

In relation to the overall prevalence of disease-related malnutrition in children, current findings are in accordance with other existing epidemiological studies, which show that the prevalence of paediatric disease related malnutrition in Europe at the time of admission is ranging from 6.1% to 25% [25,26,27]. In Greece, such data are sparse due to the lack of a routinely performed national malnutrition screening program in clinical settings. In the study by Chourdakis et al. [26], amongst 12 European countries, data from Greece were also analysed (n = 140), showing the prevalence of children at high malnutrition risk as assessed by the PYMS tools to be as high as 30%. This is almost double the percentage of our findings, and this difference could be attributed to the difference in the sample size, as in the aforementioned study, the sample size was smaller and yet not representative. 

Moreover, in the current study, the disease related malnutrition risk was found to be higher in paediatric wards compared to surgical wards. Factors that actually influence disease related malnutrition in hospitalised children seem to depend on the type of surgery they will undergo, while the presence of cardiovascular [32], GI, and orthopaedic disorders seem to be connected with higher prevalence of malnutrition in this population. [33]. According to our results, most surgical patients in the present study were subjected to minor GI surgeries, which could explain the higher percentage of disease-related malnutrition risk, observed in the paediatric wards, in which most prevalent cause of admission was gastrointestinal diseases.

The current study has some limitations. First of all, not all children included in the study referred to the clinical paediatric dietitians. It should be stressed though that no differences were identified in descriptive characteristics between those who did and those who did not receive the dietitian’s feedback. Although, the present study was conducted only in two hospitals in Greece, these are the largest hospitals that receive referrals from the all over the country, and thus, our sample could be considered as random. Furthermore, the present study used both WHO and Hellenic growth charts to evaluate the diagnostic accuracy of PYMS and STAMP, taking into account both national and international guidelines, as well as local practices for the assessment of growth in children.

## 5. Conclusions

In conclusion, PYMS [20] was found superior compared to STAMP [19], based on its sensitivity and specificity against dietitians’ global clinical judgment. Its efficacy was independent from the growth charts used (i.e., WHO or Hellenic ones) or the wards implemented (paediatric versus surgical), and thus, its use in clinical practice is recommended. Given that 15% of the hospitalised children were at high risk and 13% at medium risk of malnutrition, the nutritional screening procedure should be established as a routine in the Greek children’s hospitals to allow early detection and diagnosis of disease related malnutrition.

## Figures and Tables

**Table 1:** Descriptive characteristics of the participants.

	Whole Sample	Additional Risk Assessment by Dietitians	*p*
Characteristics	n = 1506	n = 907	
Sex (Girls), n (%)	627 (42%)	380 (41.9)	0.83
Age (years), median (IQR)	5.7 (3.0–10.5)	6.0 (3.0–10.5)	0.12
Anthropometric/dietary characteristics			
Weight (kg), median (IQR)	22.0 (14.5–39)	21.0 (14.5–39)	0.29
BMI (kg/m^2^), median (IQR)	16.6 (15.2–19.2)	16.5 (15.2–19.1)	0.68
Recent weight loss (yes), n (%)	283 (18.8)	167 (18.4)	0.54
Decreased food intake (yes), n (%)	192 (45.6)	124 (13.7)	0.20
Type of Ward			
Surgical, n (%)	638 (42)	298 (32.9)	<0.001
Paediatric, n (%)	868 (58)	609 (67.1)	
Main Causes of admission, n (%)			
Gastrointestinal disease, n (%)	391 (26)	223 (24.6)	0.12
Minor Surgeries, n (%)	358 (23.8)	154 (17.0)	<0.001
Other Causes of admission, n (%)			
Neurological disorder, n (%)	184 (12.2)	131 (14.4)	0.002
Respiratory disorder, n (%)	159 (10.6)	112 (12.3)	0.006
Fever, n (%)	144 (9.5%)	96 (10.6%)	0.13
Fall/accident/fracture, n (%)	40 (2.7%)	29 (3.2%)	0.14
Infection disease, n (%)	38 (2.5%)	25 (2.8%)	0.61
Allergy disease, n (%)	31 (2%)	24 (2.6%)	0.06
Diabetes Mellitus, n (%)	27 (1.8%)	19 (2.1%)	0.34
Oncology disorder, n (%)	24 (1.6%)	15 (1.7%)	<0.99
Other, n (%)	110 (7.3%)	35 (3.9%)	0.14
Dietetic assessment risk			
Low	-	713 (47.3)	
Medium	-	129 (8.6)	
High	-	65 (4.3)	

Data are presented as median (Interquartile range) for continuous skewed outcomes and as absolute (n) and relative frequencies for binary outcomes (%); BMI: body mass index; IQR: interquartile range; *p*-value as derived from chi-squared test for categorical variables or Mann-Whitney for skewed continuous variables. Values in bold are indicative of statistical significance defined as *p* < 0.05.

**Table 2:** Evaluation of the efficacy of nutritional screening tools to predict disease related malnutrition versus dietitian’s global clinical judgment (n = 907).

Dietetic Assessment	PYMS		STAMP	
	WHO	HGC	WHO	HGC
Sensitivity (%)	82.0	88.2	84.4	78.3
Specificity (%)	84.0	82.5	63.8	67.6
PPV (%)	58.2	57.7	38.7	39.5
NPV (%)	94.5	96.2	93.8	92
Cohen’s Kappa value (95% ΔΕ)	0.47 (0.41–0.52)	0.48 (0.43–0.53)	0.28 0.23–0.33	0.26 (0.21–0.32)

Data are presented as relative frequencies (%); Confidence interval was calculated using the formula: estimate ± 1.96 standard error; CI: confidence interval, HGC: Hellenic growth charts, NPV: negative predictive value, PPV: positive predictive value, PYMS: Paediatric Yorkhill Malnutrition Score, STAMP: Screening Tool for the Assessment of Malnutrition in Paediatrics, WHO: World Health Organization.

**Table 3:** Characteristics of children based on PYMS_WHO in the whole sample (n = 1506).

	PYMS_WHO			
	Low Risk	Medium Risk	High Risk	*p*
Variables	n = 1085 (72.0%)	n = 197 (13.1%)	n = 224 (14.9%)	
Age (years)	6.2 (3, 11) ^†^	6.2 (3, 10.5) ^†^	5 (2.5, 8.8)	0.02
Sex (girls), n (%)	418 (38.5) ^†^	105 (53.3)	104 (46.4)	<0.001
Height (cm)	1.20 (0.99, 1.46) ^†^	1.19 (0.94, 1.45) ^†^	1.11 (0.92, 1.31)	0.003
Weight (kg)	23 (15.2, 41.0) ^†^	23 (14, 38) ^†^	17.5 (12.5, 27.8)	<0.001
BMI	17.0 (15.4, 19.6) ^†^	16.5 (15.3, 18.6) ^†^	14.8 (13.2, 16.6)	<0.001
<−2 SDSs, n (%)	0	0	76 (33.9)	<0.001
<−2 SDSs and not categorized at the high risk group, n (%)	-	0	-	-
Recent Weight loss (yes), n (%)	0 ^†^	101 (51.3) ^†^	168 (75.0)	<0.001
Low food intake (yes), n (%)	0 ^†^	46 (23.4) ^†^	146 (65.2)	<0.001
Length of hospitalisation (days)	2 (2.4) ^†^	3 (2.6)	3 (2.7)	<0.001

Data are presented as median (Interquartile range) for continuous skewed outcomes and as absolute (n) and relative frequencies for binary outcomes (%); BMI: Body Mass Index; PYMS: Paediatric Yorkhill Malnutrition Score; SDS: Standard Deviation score (s); WHO: World Health Organization; *p* value as derived from Kruskal-Wallis H test for continuous skewed variables and chi-squared test for categorical variables. Comparisons between two groups were made using Mann-Whitney U test. Values in bold are indicative of statistical significance defined as *p* < 0.05; ^†^ Significant different from the high risk group (*p* < 0.05).

**Table 4:** Characteristics of children not classified as high risk from PYMS, although had increased risk according to dietetic judgments (n = 907).

	Missed Cases from PYMS_WHO ^a^	Non-Missed Cases from PYMS_WHO ^b^	*p* *
	n = 9	n = 56	
Age (years)	5.5 (2.9, 11.2)	6.0 (3.0, 10.0)	0.66
Height (cm)	1.12 (0.93, 1.45)	1.15 (0.99, 1.35)	0.30
Weight (kg)	17.2 (12.9, 31.0)	18.0 (13.0, 25.7)	0.44
BMI	14.4 (13.4, 15.4)	14.6 (13.1, 16.3)	0.85
<−2 SDSs, n (%)	0	20 (35.7)	<0.001
Recent Weight loss (yes), n (%)	1 (11.1)	44 (78.6)	<0.001
Low food intake, (yes) n (%)	3 (33.3)	36 (64.3)	<0.001
Length of hospitalisation (days)	4.0 (2.0, 11.5)	6.0 (4.0, 11.7)	0.002

Data are presented as median (Interquartile range) for continuous skewed outcomes and as absolute (n) and relative frequencies for binary outcomes (%). ^a^ Missed cases are defined those children who categorised as high risk from dietetic referrals but as low or medium risk based on PYMS either using WHO or HGC criteria. ^b^ Non-missed cases are defined those children who categorised as high risk from dietetic referrals and PYMS (either using WHO or HGC criteria) as well. * *p* value as derived from Mann-Whitney U test for continuous skewed variables and chi-squared test for categorical variables. Values in bold are indicative of statistical significance defined as *p* < 0.05.

## Supplementary Materials

The following are available online at https://www.mdpi.com/article/10.3390/nu13041279/s1, Figure S1: Prevalence of disease related malnutrition risk based on PYMS_HGC in whole sample and in Paediatric and Surgical wards (PYMS: Paediatric Yorkhill Malnutrition Score; HGC: Hellenic Growth chart), Table S1: Evaluation of the efficacy of nutritional screening tools to predict disease related malnutrition versus dietitian’s global clinical judgments, in paediatric wards and surgical wards, Table S2: Logistic regression analysis models, exploring the association between PYMS_WHO steps and predicted outcome from dietetic global clinical judgments (n = 907).
