# Supplementary material for: Malnutrition in Hospitalised Children—An Evaluation of the Efficacy of Two Nutritional Screening Tools
Source: Nutrients. 2021 Apr 13;13(4):1279. doi: 10.3390/nu13041279 (PMC8069022; doi:10.3390/nu13041279)
Supplement: Supplementary file 1 [file nutrients-13-01279-s001.pdf]

Supplementary Information

Table S1: Evaluation of the efficacy of nutritional screening tools to predict disease related malnutrition versus dietitian's global clinical judgments, in paediatric wards and in surgical wards.

Ward	Paediatric Ward				Surgical Ward			
Dietetic assessment	PYMS		STAMP		PYMS		STAMP	
	WHO	HGC	WHO	HGC	WHO	HGC	WHO	HGC
Sensitivity (%)	85.2	91	84.4	73.7	83.3	76.4	84.5	85.9
Specificity (%)	80.1	78	71	75.6	92.5	92	48.2	50.4
PPV (%)	76.4	76.9	33.9	35.2	51.7	50.9	42.2	43.1
NPV (%)	92.5	94.5	90.8	91.9	95.6	97.2	94.8	92.0
Cohen's Kappa value (95% ΔΕ)	0.42 (0.36-0.49)	0.42 (0.36-0.48)	0.32 (0.25-0.38)	0.29 (0.22-0.35)	0.58 (0.49-0.68)	0.61 (0.52-0.70)	0.21 (0.13-0.29)	0.22 (0.14-0.29)

Data are presented as relative frequencies (%).

[a]: Confidence interval was calculated using the formula: estimate±1.96 standard error.

CI: confidence interval, **HGC**: Hellenic growth charts, **NPV**: negative predictive value, **PPV**: positive predictive value, **PYMS**: Paediatric Yorkhill Malnutrition Score, **STAMP**: Screening Tool for the Assessment of Malnutrition in Paediatrics, **WHO**: World Health Organization.

Table S2: Logistic regression analysis models, exploring the association between PYMS_WHO[1] steps and predicted outcome from dietetic global clinical judgments (n=907).

	Dependent variable: Dietetic judgment (low vs. high risk)[2]		
	OR	95% CI	P[3]
Step 1: Is the BMI below the cut-off point? (yes vs. no)	14.1	7.9-25.3	<0.001
Step 2: Has the child lost weight recently? (yes vs. no)	2.2	1.3-3.7	0.002
Step 3: Has the child had a reduced intake (including feeds) for at least the past week? (yes vs. no)	11.7	6.7-20.4	<0.001
Step 4: Will the child's nutrition be affected by the recent admission/condition for at least the next week? (yes vs. no)	18.1	9.4-35	<0.001

CI: confidence interval; **OR**: odds ratio.

[1] No differences in statistical significance were observed when PYMS_HGC steps were used in the analysis.

[2] The model is adjusted for age and gender.

[3] P value as derived from the logistic regression model. Values in bold are indicative of statistical significance defined as $P<0.05$.

Figure S1: Prevalence of disease related malnutrition risk based on PYMS_HGC in whole sample and in Paediatric and Surgical ward (**PYMS**: Pediatric Yorkhill Malnutrition Score; **HGC**: Hellenic Growth chart).

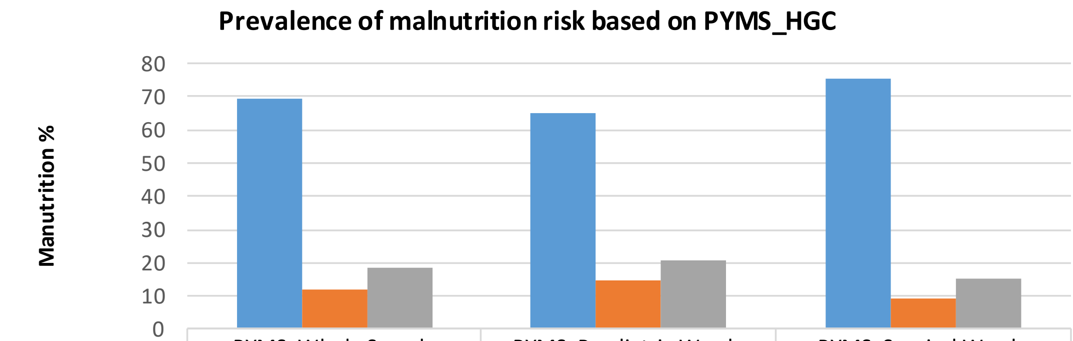

	PYMS_Whole Sample	PYMS_Paediatric Wards	PYMS_Surgical Wards
Low risk (%)	69.4	65	75.4
Medium risk (%)	12.2	14.5	9.1
High risk (%)	18.4	20.5	15.5
